# Supplementary material for: Characteristics and Health Risk Assessment of Semi-Volatile Organic Contaminants in Rural Pond Water of Hebei Province
Source: Int J Environ Res Public Health. 2019 Nov 14;16(22):4481. doi: 10.3390/ijerph16224481 (PMC6887736; doi:10.3390/ijerph16224481)
Supplement: Supplementary file 1 [file ijerph-16-04481-s001.zip › Table S/Table S1.pdf]

Table S1. Concentrations of target organic contaminants in ten pond water samples collected in Hebei province (ng/L).

	**S1**	**S2**	**S3**	**S4**	**S5**	**S6**	**S7**	**S8**	**S9**	**S10**
Compounds										
phenol	26.2	53.1	-	-	2614	-	-	71.9	60.8	-
m-cresol	-	-	-	-	1544	-	-	418	-	-
p-cresol	-	168	-	-	12860	-	-	-	-	-
o-nitrophenol	-	-	-	-	-	-	-	155	-	-
2,4-dimethylphenol	-	-	-	-	-	-	-	191	-	-
2,4-dichlorophenol	-	36.0	-	-	16.1	-	-	-	-	-
aniline	33.7	52.7	-	-	-	46.4	-	2572	-	146
p-chloroaniline	-	-	-	-	-	65.0	61.1	39.9	-	-
naphthalene	13.4	-	28.3	11.5	20.5	17.7	14.2	78.5	15.3	16.7
2-methylnaphthalene	-	-	-	-	-	-	-	82.1	-	-
1-methylnaphthalene	-	-	-	-	-	-	-	92.0	-	-
1,3-dimethylnaphthalene	-	-	-	-	-	-	-	40.4	-	-
acenaphthylene	34.5	-	-	5.07	-	-	-	-	6.92	6.38
1-aminonaphthalene	826	38.1	-	-	-	-	52.5	-	28.7	-
2-aminonaphthalene	737	120	-	-	-	-	243	-	69.6	-
fluorene	-	3.98	-	-	-	-	-	-	-	-
phenanthrene	-	22.0	-	-	80.6	31.1	23.7	35.6	-	-
fluoranthene	-	17.3	7.03	5.42	52.1	9.26	7.07	10.1	4.29	5.91
pyrene	-	20.9	7.37	6.48	54.6	24.2	14.5	-	8.26	7.75
benzo[a]anthracene	9.46	27.6	5.04	5.45	42.5	36.4	19.1	8.27	7.55	7.53
chrysene	14.4	50.8	-	-	68.2	52.1	29.2	7.11	8.24	8.03
benzo[b]fluoranthene	9.24	29.9	-	-	51.0	37.2	19.2	7.82	7.95	9.76
benzo[k]fluoranthene	6.98	29.4	-	-	-	37.3	17.2	4.28	4.24	-

dimethyl phthalate	-	-	-	-	-	-	59	2170	-	-
diisobutyl phthalate	-	-	-	-	-	-	-	630	-	-
dibutoxyethyl phthalate	-	-	-	-	350	-	-	-	-	-
di-(2-ethylhexyl) phthalate	695	-	580	720	960	-	-	-	-	-
sum	2406	670	628	754	18713	357	560	6614	222	208

"-" represents below the detection limit. Undetected contaminants were not included.
