# Supplementary material for: Characteristics and Health Risk Assessment of Semi-Volatile Organic Contaminants in Rural Pond Water of Hebei Province
Source: Int J Environ Res Public Health. 2019 Nov 14;16(22):4481. doi: 10.3390/ijerph16224481 (PMC6887736; doi:10.3390/ijerph16224481)
Supplement: Supplementary file 1 [file ijerph-16-04481-s001.zip › Table S/Table S2.pdf]

Table S2. The hazard quotients (HQ) and hazard index (HI) for noncarcinogenic risk through two exposure pathways in each rural pond water sample.

Compounds	Non-carcinogenic risk									
	Direct ingestion									
	**S1**	**S2**	**S3**	**S4**	**S5**	**S6**	**S7**	**S8**	**S9**	**S10**
phenol	2.91E-06	5.90E-06	-	-	2.90E-04	-	-	7.99E-06	6.75E-06	-
m-cresol	-	-	-	-	1.03E-03	-	-	2.79E-04	-	-
p-cresol	-	5.60E-05	-	-	4.29E-03	-	-	-	-	-
2,4-dimethylphenol	-	-	-	-	-	-	-	3.18E-04	-	-
2,4-dichlorophenol	-	4.00E-04	-	-	1.79E-04	-	-	-	-	-
aniline	1.61E-04	2.51E-04	-	-	-	2.21E-04	-	1.22E-02	-	6.94E-04
p-chloroaniline	-	-	-	-	-	5.42E-04	5.09E-04	3.33E-04	-	-
naphthalene	2.23E-05	-	4.71E-05	1.91E-05	3.42E-05	2.96E-05	2.36E-05	1.31E-04	2.56E-05	2.78E-05
2-methylnaphthalene	-	-	-	-	-	-	-	6.84E-04	-	-
1-methylnaphthalene	-	-	-	-	-	-	-	4.38E-05	-	-
fluorene	-	3.32E-06	-	-	-	-	-	-	-	-
fluoranthene	-	1.44E-05	5.86E-06	4.52E-06	4.34E-05	7.71E-06	5.89E-06	8.46E-06	3.58E-06	4.92E-06
pyrene	-	2.33E-05	8.19E-06	7.20E-06	6.06E-05	2.69E-05	1.62E-05	-	9.18E-06	8.61E-06
di-(2-ethylhexyl) phthalate	1.16E-03	-	9.67E-04	1.20E-03	1.60E-03	-	-	-	-	-
HI	1.34E-03	7.54E-04	1.03E-03	1.23E-03	7.52E-03	8.27E-04	5.55E-04	1.41E-02	4.51E-05	7.35E-04

"-" represents no values

Compounds	Non-carcinogenic risk									
	Dermal absorption									
	S1	S2	S3	S4	S5	S6	S7	S8	S9	S10
phenol	2.00E-08	4.06E-08	-	-	2.00E-06	-	-	5.50E-08	4.64E-08	-
m-cresol	-	-	-	-	1.28E-05	-	-	3.47E-06	-	-
p-cresol	-	6.76E-07	-	-	5.17E-05	-	-	-	-	-
2,4-dimethylphenol	-	-	-	-	-	-	-	5.54E-06	-	-
2,4-dichlorophenol	-	1.32E-05	-	-	5.91E-06	-	-	-	-	-
aniline	4.88E-07	7.63E-07	-	-	-	6.72E-07	-	3.72E-05	-	2.11E-06
p-chloroaniline	-	-	-	-	-	4.30E-06	4.04E-06	2.64E-06	-	-
naphthalene	1.67E-06	-	3.51E-06	1.43E-06	2.55E-06	2.21E-06	1.76E-06	9.76E-06	1.91E-06	2.08E-06
2-methylnaphthalene	-	-	-	-	-	-	-	1.00E-04	-	-
1-methylnaphthalene	-	-	-	-	-	-	-	6.53E-06	-	-
fluorene	-	5.84E-07	-	-	-	-	-	-	-	-
fluoranthene	-	7.09E-06	2.89E-06	2.23E-06	2.14E-05	3.80E-06	2.90E-06	4.17E-06	1.76E-06	2.43E-06
pyrene	-	7.49E-06	2.63E-06	2.32E-06	1.95E-05	8.65E-06	5.20E-06	-	2.95E-06	2.77E-06
di-(2-ethylhexyl) phthalate	2.09E-03	-	1.75E-03	2.17E-03	2.89E-03	-	-	-	-	-
HI	2.10E-03	2.98E-05	1.76E-03	2.18E-03	3.01E-03	1.96E-05	1.39E-05	1.70E-04	6.67E-06	9.38E-06

"-" represents no values
