# Supplementary material for: Characteristics and Health Risk Assessment of Semi-Volatile Organic Contaminants in Rural Pond Water of Hebei Province
Source: Int J Environ Res Public Health. 2019 Nov 14;16(22):4481. doi: 10.3390/ijerph16224481 (PMC6887736; doi:10.3390/ijerph16224481)
Supplement: Supplementary file 1 [file ijerph-16-04481-s001.zip › Table S/Table S3.pdf]

Table S3. The hazard quotients (HQ) and total risk (∑R) for carcinogenic risk through two exposure pathways in each rural pond water sample.

Compounds	Carcinogenic risk									
	Direct ingestion									
	**S1**	**S2**	**S3**	**S4**	**S5**	**S6**	**S7**	**S8**	**S9**	**S10**
aniline	6.41E-09	1.00E-08	-	-	-	8.83E-09	-	4.89E-07	-	2.77E-08
p-chloroaniline	-	-	-	-	-	4.34E-07	4.08E-07	2.66E-07	-	-
1-methylnaphthalene	-	-	-	-	-	-	-	8.89E-08	-	-
benzo[a]anthracene	3.15E-08	9.20E-08	1.68E-08	1.82E-08	1.42E-07	1.21E-07	6.38E-08	2.76E-08	2.52E-08	2.51E-08
chrysene	4.79E-10	1.69E-09	-	-	2.27E-09	1.74E-09	9.72E-10	2.37E-10	2.75E-10	2.68E-10
benzo[b]fluoranthene	3.08E-08	9.97E-08	-	-	1.70E-07	1.24E-07	6.40E-08	2.61E-08	2.65E-08	3.25E-08
benzo[k]fluoranthene	2.33E-09	9.81E-09	-	-	-	1.24E-08	5.73E-09	1.43E-09	1.41E-09	-
di-(2-ethylhexyl) phthalate	3.24E-07	-	2.71E-07	3.36E-07	4.48E-07	-	-	-	-	-
∑R	3.96E-07	2.13E-07	2.87E-07	3.54E-07	7.62E-07	7.02E-07	5.42E-07	8.99E-07	5.34E-08	8.56E-08

"-" represents no values.

Compounds	Carcinogenic risk									
	Dermal absorption									
	**S1**	**S2**	**S3**	**S4**	**S5**	**S6**	**S7**	**S8**	**S9**	**S10**
aniline	1.95E-11	3.04E-11	-	-	-	2.68E-11	-	1.49E-09	-	8.41E-11
p-chloroaniline	-	-	-	-	-	3.44E-09	3.23E-09	2.11E-09	-	-
1-methylnaphthalene	-	-	-	-	-	-	-	1.32E-08	-	-
benzo[a]anthracene	2.79E-08	8.13E-08	1.49E-08	1.60E-08	1.25E-07	1.07E-07	5.63E-08	2.44E-08	2.22E-08	2.22E-08
chrysene	4.56E-10	1.61E-09	-	-	2.17E-09	1.66E-09	9.27E-10	2.26E-10	2.62E-10	2.55E-10
benzo[b]fluoranthene	2.06E-08	6.65E-08	-	-	1.13E-07	8.27E-08	4.27E-08	1.74E-08	1.77E-08	2.17E-08

benzo[k]fluoranthene	2.57E-09	1.08E-08	-	-	-	1.38E-08	6.33E-09	1.58E-09	1.56E-09	-
di-(2-ethylhexyl) phthalate	5.86E-07	-	4.89E-07	6.07E-07	8.10E-07	-	-	-	-	-
∑R	6.38E-07	1.60E-07	5.04E-07	6.24E-07	1.05E-06	2.09E-07	1.09E-07	6.04E-08	4.17E-08	4.42E-08

"-" represents no values
